# Supplementary material for: An open-source framework for large-scale, flexible evaluation of biomedical text mining systems
Source: J Biomed Discov Collab. 2008 Jan 29;3:1. doi: 10.1186/1747-5333-3-1 (PMC2276192; doi:10.1186/1747-5333-3-1)
Supplement: Additional file 1 — Evaluation platform source code. The additional file contains the software evaluation framework discussed in this paper. The most current release can be downloaded from SourceForge.net [1]. See the accompanying README file for instructions on installation and use. Also included in the distribution is a collection of UIMA wrappers for some commonly used BioNLP tools and annotated corpora. [file 1747-5333-3-1-S1.zip › bionlp_uima_repository-1.0.1/docs/api/edu/uchsc/ccp/util/xml/XMLValidityChecker.html]

XMLValidityChecker (Center for Computational Pharmacology Util/UIMA API)


|  |  |  |
| --- | --- | --- |
| **Overview** \| **Package** \| **Class** \| **Tree** \| **Deprecated** \| **Index** \| **Help** | |  |
| PREV CLASS   NEXT CLASS | **FRAMES**    **NO FRAMES**     **All Classes** |  |
| SUMMARY: NESTED \| FIELD \| CONSTR \| METHOD | DETAIL: FIELD \| CONSTR \| METHOD |  |


---


## edu.uchsc.ccp.util.xml Class XMLValidityChecker

```
java.lang.Object
  edu.uchsc.ccp.util.xml.XMLValidityChecker
```

---

``` public class XMLValidityChecker extends Object ```

This class utilizes the XMLReader class to validate XML.

The code in this class was modified from: http://www.cafeconleche.org/books/xmljava/chapters/ch09s06.html

**Author:**
:   William A Baumgartner, Jr.

---

**Constructor Summary**

`XMLValidityChecker()`


| **Method Summary** | |
| --- | --- |
| `static boolean` | `validateXML(String xmlFile)`             Validates the input XML file. |

**Methods inherited from class java.lang.Object**

`clone, equals, finalize, getClass, hashCode, notify, notifyAll, toString, wait, wait, wait`

**Constructor Detail**

### XMLValidityChecker

```
public XMLValidityChecker()
```


**Method Detail**

### validateXML

```
public static boolean validateXML(String xmlFile)
```

:   Validates the input XML file.

    :   **Parameters:**: `xmlFile` - XML file to be validated **Returns:**: true is XML is valid, false otherwise


---


|  |  |  |
| --- | --- | --- |
| **Overview** \| **Package** \| **Class** \| **Tree** \| **Deprecated** \| **Index** \| **Help** | |  |
| PREV CLASS   NEXT CLASS | **FRAMES**    **NO FRAMES**     **All Classes** |  |
| SUMMARY: NESTED \| FIELD \| CONSTR \| METHOD | DETAIL: FIELD \| CONSTR \| METHOD |  |


---
